# Supplementary material for: Tracking down the White Plague. Chapter three: Revision of endocranial abnormally pronounced digital impressions as paleopathological diagnostic criteria for tuberculous meningitis
Source: PLoS One. 2021 Mar 19;16(3):e0249020. doi: 10.1371/journal.pone.0249020 (PMC7978373; doi:10.1371/journal.pone.0249020)
Supplement: S4 Table — (TB = tuberculosis; NTB = non-tuberculous; TBM = tuberculous meningitis; APDIs = abnormally pronounced digital impressions; ABVIs = abnormal blood vessel impressions; PAs = periosteal appositions; GIs = granular impressions; + = present;– = not present). (PDF) [file pone.0249020.s004.pdf]

S4 Table: Individual data of cases exhibiting APDIs regarding other probable TBM-related endocranial alterations in the NTB group (Σ=62). (TB = tuberculosis; NTB = non-tuberculous; TBM = tuberculous meningitis; APDIs = abnormally pronounced digital impressions; ABVIs = abnormal blood vessel impressions; PAs = periosteal appositions; GIs = granular impressions; + = present; – = not present)

No.	Terry No.	APDIs	ABVIs	PAs	GIs
1	19R	+	−	−	−
2	58R	+	−	+	−
3	62RR	+	−	−	−
4	64R	+	−	−	−
5	112R	+	−	−	−
6	127R	+	+	−	−
7	135R	+	−	−	−
8	167	+	−	−	−
9	197R	+	−	−	+
10	209	+	−	−	−
11	218	+	−	−	−
12	227	+	−	−	−
13	231	+	+	−	−
14	259	+	−	−	−
15	268	+	−	−	−
16	272	+	−	+	+
17	296R	+	−	−	−
18	314	+	−	−	−
19	339R	+	−	−	−
20	348R	+	−	−	−
21	403	+	−	−	−
22	422	+	−	−	−
23	437R	+	−	−	−
24	445	+	−	−	−
25	447	+	−	−	−
26	453	+	−	−	−
27	463	+	−	−	−
28	465	+	−	−	+
29	470	+	−	+	−
30	483	+	−	−	−
31	496	+	−	−	−
32	506	+	−	−	+
33	512	+	+	−	−
34	536	+	−	+	−
35	545	+	−	−	−
36	573	+	−	−	−

No.	Terry No.	APDIs	ABVIs	PAs	GIs
37	597	+	−	−	−
38	636	+	−	−	−
39	657R	+	−	−	−
40	694	+	−	−	−
41	759	+	−	+	−
42	795	+	−	−	−
43	823	+	−	−	−
44	833R	+	−	−	−
45	946	+	−	+	−
46	973	+	−	−	−
47	1023	+	−	−	−
48	1046	+	−	−	−
49	1130R	+	−	−	−
50	1138R	+	−	−	−
51	1140	+	−	−	−
52	1186	+	−	−	−
53	1192	+	−	−	−
54	1224	+	−	+	−
55	1228	+	−	−	−
56	1229	+	−	−	−
57	1291	+	−	−	−
58	1342	+	−	−	−
59	1417R	+	−	−	−
60	1505R	+	−	−	−
61	1599	+	−	−	−
62	1614	+	−	−	−
