# Supplementary material for: Tracking down the White Plague. Chapter three: Revision of endocranial abnormally pronounced digital impressions as paleopathological diagnostic criteria for tuberculous meningitis
Source: PLoS One. 2021 Mar 19;16(3):e0249020. doi: 10.1371/journal.pone.0249020 (PMC7978373; doi:10.1371/journal.pone.0249020)
Supplement: S5 Table — (TB = tuberculosis; APDIs = abnormally pronounced digital impressions; PNBFs = periosteal new bone formations; HPO = hypertrophic pulmonary osteopathy; + = present;– = not present). (PDF) [file pone.0249020.s005.pdf]

S5 Table: Individual data of cases exhibiting APDIs on the inner skull surface regarding probable TB-related non-endocranial lesions in the TB group (Σ=154). (TB = tuberculosis; APDIs = abnormally pronounced digital impressions; PNBFs = periosteal new bone formations; HPO = hypertrophic pulmonary osteopathy; + = present; – = not present)

No.	Terry No.	APDIs	PNBFs on the visceral costal surfaces	HPO	Extra-spinal osteomyelitis	Extra-spinal arthritis	Vertebral hypervascularization	Vertebral lytic lesions and/or arthritis	Reactive new bone formations indicative of a cold abscess
1	13R	+	+	–	–	–	+	–	–
2	30R	+	–	–	–	–	+	–	–
3	54	+	–	–	–	–	–	–	+
4	84	+	–	–	–	–	–	+	+
5	89R	+	+	–	–	–	+	+	+
6	90	+	+	–	–	–	+	–	–
7	95	+	–	+	–	–	–	+	+
8	95R	+	+	–	–	–	+	–	–
9	128	+	+	–	–	–	+	–	–
10	129	+	+	–	+	–	+	+	+
11	138	+	+	–	–	–	–	–	+
12	139	+	–	–	–	–	+	–	–

No.	Terry No.	APDIs	PNBFs on the visceral costal surfaces	HPO	Extra-spinal osteomyelitis	Extra-spinal arthritis	Vertebral hypervascularization	Vertebral lytic lesions and/or arthritis	Reactive new bone formations indicative of a cold abscess
13	146R	+	−	−	−	−	−	−	−
14	182	+	+	−	−	−	+	−	+
15	194	+	−	−	−	−	−	−	−
16	204	+	+	−	−	+	+	−	−
17	205	+	−	−	−	+	+	−	−
18	207	+	+	−	−	+	−	−	−
19	220	+	−	−	−	−	−	−	−
20	222	+	+	−	−	−	+	−	−
21	230	+	+	−	−	−	−	−	−
22	235	+	−	−	−	−	+	−	+
23	251	+	+	−	−	−	−	−	−
24	254	+	+	+	−	−	−	−	−
25	255	+	+	−	−	−	+	−	−
26	265	+	+	−	−	−	+	+	+

No.	Terry No.	APDIs	PNBFs on the visceral costal surfaces	HPO	Extra-spinal osteomyelitis	Extra-spinal arthritis	Vertebral hypervascularization	Vertebral lytic lesions and/or arthritis	Reactive new bone formations indicative of a cold abscess
27	267	+	−	−	+	−	+	−	−
28	269	+	+	−	−	−	+	−	+
29	270	+	−	−	−	−	+	−	−
30	279	+	+	−	−	−	+	+	−
31	280	+	−	−	−	−	+	−	−
32	283R	+	−	−	−	−	+	−	−
33	304	+	−	−	−	−	+	−	−
34	306	+	+	+	−	−	+	−	−
35	341	+	−	−	−	−	−	−	−
36	382R	+	+	−	−	−	+	−	−
37	385	+	−	−	−	−	+	−	−
38	400	+	+	−	−	−	+	−	−
39	423	+	+	−	−	−	+	−	−
40	466	+	+	−	−	−	+	+	−

No.	Terry No.	APDIs	PNBFs on the visceral costal surfaces	HPO	Extra-spinal osteomyelitis	Extra-spinal arthritis	Vertebral hypervascularization	Vertebral lytic lesions and/or arthritis	Reactive new bone formations indicative of a cold abscess
41	468	+	+	−	+	+	+	+	+
42	522	+	+	−	−	−	−	−	−
43	562	+	+	−	−	−	+	−	−
44	565	+	+	−	−	−	+	−	−
45	572	+	+	−	−	−	−	−	−
46	583	+	−	−	−	−	+	−	−
47	585	+	−	−	−	−	−	−	−
48	592	+	+	+	+	−	+	−	−
49	595	+	−	−	−	−	+	−	−
50	621R	+	+	−	−	−	+	−	−
51	664	+	+	−	−	−	+	−	−
52	728R	+	−	−	−	−	+	−	−
53	752	+	−	−	−	−	−	−	−
54	757	+	−	−	−	−	−	−	−

No.	Terry No.	APDIs	PNBFs on the visceral costal surfaces	HPO	Extra-spinal osteomyelitis	Extra-spinal arthritis	Vertebral hypervascularization	Vertebral lytic lesions and/or arthritis	Reactive new bone formations indicative of a cold abscess
55	771	+	−	−	−	−	−	−	−
56	776	+	−	−	−	+	+	−	−
57	786	+	−	−	−	−	+	−	−
58	799	+	+	−	−	−	+	−	−
59	820R	+	+	−	−	−	−	−	−
60	822	+	+	−	−	−	+	−	−
61	828	+	+	+	−	−	−	−	−
62	844	+	−	−	−	−	−	−	−
63	846	+	+	−	−	−	+	−	−
64	876	+	+	−	−	−	+	−	−
65	892	+	+	−	−	−	+	−	−
66	896RR	+	+	−	−	+	+	−	−
67	897	+	+	−	−	−	+	+	−
68	907	+	−	−	−	−	−	−	−

No.	Terry No.	APDIs	PNBFs on the visceral costal surfaces	HPO	Extra-spinal osteomyelitis	Extra-spinal arthritis	Vertebral hypervascularization	Vertebral lytic lesions and/or arthritis	Reactive new bone formations indicative of a cold abscess
69	914	+	−	−	−	−	−	−	−
70	915	+	+	−	−	−	+	−	−
71	932	+	−	−	−	−	−	−	−
72	933R	+	+	−	−	−	+	−	−
73	936	+	−	−	−	−	+	−	−
74	950	+	+	−	−	−	−	−	−
75	955	+	+	+	−	−	+	−	−
76	987	+	+	+	−	−	−	−	−
77	1002	+	−	−	−	−	−	−	−
78	1005	+	−	−	−	−	−	−	−
79	1013	+	+	−	−	−	+	−	−
80	1018	+	+	−	+	−	+	+	+
81	1020	+	+	+	−	−	+	−	−
82	1031	+	−	−	−	−	+	−	−

No.	Terry No.	APDIs	PNBFs on the visceral costal surfaces	HPO	Extra-spinal osteomyelitis	Extra-spinal arthritis	Vertebral hypervascularization	Vertebral lytic lesions and/or arthritis	Reactive new bone formations indicative of a cold abscess
83	1033	+	+	−	−	−	+	−	−
84	1034	+	−	−	−	−	−	−	−
85	1036	+	−	−	−	−	+	−	−
86	1047	+	+	−	−	−	−	−	−
87	1048	+	−	−	−	−	−	−	−
88	1057	+	+	−	−	−	−	−	−
89	1072	+	−	−	−	−	−	−	−
90	1076	+	+	−	−	−	+	−	−
91	1093	+	+	−	−	−	+	+	+
92	1095	+	−	−	−	−	−	−	−
93	1096R	+	+	+	−	−	+	−	−
94	1105	+	+	+	−	−	+	−	−
95	1106	+	+	−	+	+	−	+	+
96	1113	+	−	−	−	−	−	−	−

No.	Terry No.	APDIs	PNBFs on the visceral costal surfaces	HPO	Extra-spinal osteomyelitis	Extra-spinal arthritis	Vertebral hypervascularization	Vertebral lytic lesions and/or arthritis	Reactive new bone formations indicative of a cold abscess
97	1122	+	+	+	−	−	−	−	−
98	1129	+	+	−	−	−	−	−	−
99	1132	+	−	−	−	−	−	−	−
100	1156	+	+	+	−	−	−	−	−
101	1157	+	−	−	−	−	+	−	−
102	1159	+	+	+	−	−	+	−	−
103	1165	+	−	−	−	−	+	−	−
104	1169	+	+	−	−	−	+	−	−
105	1173	+	−	−	−	−	+	−	−
106	1183	+	+	−	−	−	+	−	−
107	1185	+	+	+	+	+	+	−	+
108	1187	+	+	−	−	−	+	−	−
109	1190	+	+	−	−	−	−	−	−
110	1205	+	+	−	−	+	−	−	−

No.	Terry No.	APDIs	PNBFs on the visceral costal surfaces	HPO	Extra-spinal osteomyelitis	Extra-spinal arthritis	Vertebral hypervascularization	Vertebral lytic lesions and/or arthritis	Reactive new bone formations indicative of a cold abscess
111	1210	+	+	−	−	−	+	−	−
112	1215	+	+	−	−	−	+	−	+
113	1222	+	−	−	−	−	−	−	−
114	1230	+	+	−	−	−	−	−	−
115	1236	+	−	−	−	−	+	−	−
116	1249R	+	−	−	−	−	−	−	−
117	1255	+	−	−	−	−	−	+	+
118	1263R	+	+	−	−	−	−	−	−
119	1264	+	−	−	−	−	−	−	−
120	1275	+	+	−	−	−	−	−	−
121	1278	+	−	−	−	−	−	−	−
122	1285	+	−	−	+	+	−	+	−
123	1287	+	+	−	−	−	+	+	+
124	1300	+	+	−	−	−	+	−	−

No.	Terry No.	APDIs	PNBFs on the visceral costal surfaces	HPO	Extra-spinal osteomyelitis	Extra-spinal arthritis	Vertebral hypervascularization	Vertebral lytic lesions and/or arthritis	Reactive new bone formations indicative of a cold abscess
125	1304R	+	−	−	−	−	−	−	−
126	1309	+	+	−	+	−	−	+	+
127	1313	+	−	−	−	−	−	−	−
128	1315	+	+	+	−	−	+	+	−
129	1318	+	+	−	−	−	−	−	−
130	1319	+	+	−	−	−	+	−	−
131	1322	+	+	−	−	−	+	−	−
132	1359	+	−	−	−	−	−	−	−
133	1362	+	+	−	−	−	+	−	−
134	1367	+	−	−	−	−	−	−	−
135	1369	+	+	−	−	−	+	−	−
136	1388	+	+	−	−	−	+	−	−
137	1397	+	+	−	−	−	−	−	−
138	1398	+	+	−	−	−	+	−	−

No.	Terry No.	APDIs	PNBFs on the visceral costal surfaces	HPO	Extra-spinal osteomyelitis	Extra-spinal arthritis	Vertebral hypervascularization	Vertebral lytic lesions and/or arthritis	Reactive new bone formations indicative of a cold abscess
139	1407	+	+	−	+	−	+	+	+
140	1422R	+	+	−	−	−	−	−	+
141	1428R	+	+	−	−	−	+	−	−
142	1434R	+	+	−	−	−	+	−	−
143	1455	+	−	−	−	−	−	−	−
144	1458	+	+	−	−	−	−	−	−
145	1503	+	+	−	−	−	+	−	−
146	1507	+	+	−	−	−	+	−	−
147	1521	+	+	−	−	−	−	−	−
148	1533	+	+	−	−	−	−	−	−
149	1539	+	+	−	−	−	−	−	−
150	1551	+	−	−	−	−	+	−	−
151	1553	+	−	−	−	−	+	−	−
152	1555	+	+	+	−	−	−	−	−

No.	Terry No.	APDIs	PNBFs on the visceral costal surfaces	HPO	Extra-spinal osteomyelitis	Extra-spinal arthritis	Vertebral hypervascularization	Vertebral lytic lesions and/or arthritis	Reactive new bone formations indicative of a cold abscess
**153**	**1562**	+	–	–	–	–	–	–	–
**154**	**1572**	+	+	–	–	–	–	–	–
