# Supplementary material for: Tracking down the White Plague. Chapter three: Revision of endocranial abnormally pronounced digital impressions as paleopathological diagnostic criteria for tuberculous meningitis
Source: PLoS One. 2021 Mar 19;16(3):e0249020. doi: 10.1371/journal.pone.0249020 (PMC7978373; doi:10.1371/journal.pone.0249020)
Supplement: S6 Table — (TB = tuberculosis; NTB = non-tuberculous; APDIs = abnormally pronounced digital impressions; PNBFs = periosteal new bone formations; HPO = hypertrophic pulmonary osteopathy; + = present;– = not present). (PDF) [file pone.0249020.s006.pdf]

S6 Table: Individual data of cases exhibiting APDIs on the inner skull surface regarding probable TB-related non-endocranial lesions in the NTB group (Σ=62).
(TB = tuberculosis; NTB = non-tuberculous; APDIs = abnormally pronounced digital impressions; PNBFs = periosteal new bone formations;
HPO = hypertrophic pulmonary osteopathy; + = present; – = not present)

No.	Terry No.	APDIs	PNBFs on the visceral costal surfaces	HPO	Extra-spinal osteomyelitis	Extra-spinal arthritis	Vertebral hypervascularization	Vertebral lytic lesions and/or arthritis	Reactive new bone formations indicative of a cold abscess
1	19R	+	–	–	–	–	+	+	–
2	58R	+	–	–	–	–	–	–	–
3	62RR	+	–	–	–	–	+	–	–
4	64R	+	–	–	–	–	–	–	–
5	112R	+	–	+	–	–	–	–	+
6	127R	+	–	–	–	–	–	–	–
7	135R	+	–	–	–	–	–	–	–
8	167	+	–	–	–	–	–	–	–
9	197R	+	–	–	–	–	–	–	–
10	209	+	–	–	–	–	+	–	–
11	218	+	+	–	–	–	+	–	–
12	227	+	–	–	–	–	–	–	–

No.	Terry No.	APDIs	PNBFs on the visceral costal surfaces	HPO	Extra-spinal osteomyelitis	Extra-spinal arthritis	Vertebral hypervascularization	Vertebral lytic lesions and/or arthritis	Reactive new bone formations indicative of a cold abscess
13	231	+	−	−	−	−	−	−	−
14	259	+	−	−	−	−	+	−	−
15	268	+	−	−	−	−	−	−	−
16	272	+	−	−	−	−	−	−	−
17	296R	+	−	−	−	−	−	−	−
18	314	+	−	−	−	−	−	−	−
19	339R	+	−	+	−	−	−	−	−
20	348R	+	−	−	−	−	−	−	−
21	403	+	−	−	−	−	−	−	−
22	422	+	−	−	−	−	−	−	−
23	437R	+	−	−	−	−	−	−	−
24	445	+	−	−	−	−	−	−	−
25	447	+	−	−	−	−	−	−	−
26	453	+	−	−	−	−	−	−	−

No.	Terry No.	APDIs	PNBFs on the visceral costal surfaces	HPO	Extra-spinal osteomyelitis	Extra-spinal arthritis	Vertebral hypervascularization	Vertebral lytic lesions and/or arthritis	Reactive new bone formations indicative of a cold abscess
27	463	+	−	−	−	−	−	−	−
28	465	+	+	−	+	−	+	−	−
29	470	+	−	−	−	−	−	−	−
30	483	+	−	−	−	−	−	−	−
31	496	+	−	−	−	−	−	−	−
32	506	+	+	+	+	−	−	−	−
33	512	+	−	−	−	−	−	−	−
34	536	+	−	−	−	−	−	−	−
35	545	+	−	−	−	−	−	−	−
36	573	+	−	−	−	−	+	−	−
37	597	+	−	−	−	−	−	−	−
38	636	+	−	−	−	−	−	−	−
39	657R	+	−	−	−	−	+	−	−
40	694	+	−	−	−	−	−	−	−

No.	Terry No.	APDIs	PNBFs on the visceral costal surfaces	HPO	Extra-spinal osteomyelitis	Extra-spinal arthritis	Vertebral hypervascularization	Vertebral lytic lesions and/or arthritis	Reactive new bone formations indicative of a cold abscess
41	759	+	−	−	−	−	−	−	−
42	795	+	−	−	−	−	+	−	−
43	823	+	−	−	−	−	−	+	−
44	833R	+	+	+	+	−	−	+	−
45	946	+	−	−	−	−	+	−	−
46	973	+	−	−	−	−	−	−	−
47	1023	+	−	−	−	−	+	−	−
48	1046	+	−	−	−	−	−	−	−
49	1130R	+	−	−	−	−	−	−	−
50	1138R	+	−	−	−	−	−	−	−
51	1140	+	−	−	−	−	−	−	−
52	1186	+	−	−	−	−	−	−	−
53	1192	+	−	−	−	−	−	−	−
54	1224	+	−	−	−	−	−	−	−

No.	Terry No.	APDIs	PNBFs on the visceral costal surfaces	HPO	Extra-spinal osteomyelitis	Extra-spinal arthritis	Vertebral hypervascularization	Vertebral lytic lesions and/or arthritis	Reactive new bone formations indicative of a cold abscess
55	1228	+	−	−	−	−	−	−	−
56	1229	+	−	−	−	−	−	−	−
57	1291	+	−	−	−	−	−	−	−
58	1342	+	−	−	−	−	−	−	−
59	1417R	+	−	+	−	−	−	−	−
60	1505R	+	−	−	−	−	−	−	−
61	1599	+	−	−	−	−	−	−	−
62	1614	+	−	−	−	−	+	−	+
